# ATF4 promotes renal tubulointerstitial fibrosis through hexokinase II-mediated glycolysis

**DOI:** 10.3389/fimmu.2025.1683249

**Published:** 2025-12-17

**Authors:** Songtao Feng, Yueming Gao, Zheng Wang, Weijie Ni, Yan Zhou, Mingyue Yuan, Jinyang Ge, Lu Sun, Bicheng Liu, Hui Qian, Zuolin Li

**Affiliations:** 1 Institute of Urinary System Diseases, Department of Nephrology, The Affiliated People’s Hospital, Jiangsu University, Zhenjiang, China; 2 Department of Basic Medicine, School of Medicine, Jiangsu University, Zhenjiang, Jiangsu, China; 3 Department of Nephrology, Peking University Third Hospital, Beijing, China; 4 Institute of Nephrology, Zhongda Hospital, Southeast University School of Medicine, Nanjing, Jiangsu, China; 5 Department of Endocrinology, The Affiliated People’s Hospital, Jiangsu University, Zhenjiang, Jiangsu, China; 6 Jiangsu Key Laboratory of Medical Science and Laboratory Medicine, Department of Laboratory Medicine, School of Medicine, Jiangsu University, Zhenjiang, Jiangsu, China

**Keywords:** kidney, tubulointerstitial fibrosis, activating transcription factor 4, hexokinase II, glycolysis

## Abstract

**Background:**

Renal tubulointerstitial fibrosis is a reliable predictor of progressive chronic kidney disease (CKD). Activating transcription factor 4 (ATF4) has recently emerged as a pivotal player in multiple pathophysiologic processes, particularly the stress response processes. This study aims to explore the role of ATF4 in tubulointerstitial fibrosis from a metabolic perspective.

**Methods:**

A murine model of renal fibrosis was generated via unilateral ureteral obstruction (UUO). Quantitative PCR was employed to assess the expression of inflammation-related genes and fibrotic markers in renal tissue, while Western blotting was used to quantify the corresponding protein levels. Immunohistochemistry was performed to determine the localization and expression patterns of ATF4. Lentivirus-mediated ATF4 knockdown mice, along with mice subjected to glycolytic inhibition, were subsequently employed to further investigate their effects on inflammatory mediators and fibrotic markers. In parallel, human renal proximal tubule epithelial cells (HK-2) were exposed to transforming growth factor-β1 (TGF-β1) to induce fibrosis *in vitro*. Subsequent molecular assays were performed to confirm the regulatory relationship between ATF4 and hexokinase II (HK-II), including verification of ATF4 binding to the HK2 promoter.

**Results:**

Western blotting and PCR analyses revealed a pronounced elevation of inflammatory cytokines, fibrotic markers, and ATF4 in the renal tissues of UUO mice compared with sham controls. Both *in vitro* and *in vivo*, ATF4 knockdown markedly mitigated tubular epithelial injury and fibrosis. Moreover, HK-II mRNA levels were significantly elevated in UUO renal tissues and in HK-2 cells stimulated with TGF-β1. Glycolytic inhibition effectively ameliorated tubular epithelial injury and fibrosis.

**Conclusion:**

In this study, we identified a marked induction of tubular ATF4 in mice subjected to UUO. Silencing ATF4 significantly mitigated renal tubulointerstitial fibrosis. Mechanistically, ATF4 was shown to act as a key glycolytic driver by transcriptionally upregulating hexokinase II. Collectively, these findings indicate that tubular ATF4 facilitates renal tubulointerstitial fibrosis through HK-II mediated glycolytic activation.

## 1 Introduction

Chronic kidney disease (CKD) has been recognized as a major public health issue affecting millions globally (1). Histologically, renal tubulointerstitial fibrosis is the main pathological characteristic of CKD (2, 3). More interestingly, emerging evidence indicated that irrespective of the initial causes, renal tubulointerstitial fibrosis is a reliable predictor of progressive CKD (4, 5). However, the molecular mechanisms of renal tubulointerstitial fibrosis progression remain poorly understood. Elucidating its molecular mechanism not only represents novel insights into the pathogenesis of CKD, but also provides a promising therapeutic targeting for delaying the progression of CKD.

Activating transcription factor 4 (ATF4), a member of the ATF/cAMP response element-binding protein family, has recently emerged as a pivotal player in multiple pathophysiologic processes, particularly the stress response processes (6, 7). Accordingly, when exposed to hypoxia, ischemia, oxidation, inflammation or metabolic insult, ATF4 undergoes rapid translational activation to begin adaptation to the acute stress (8–10). More interestingly, the timing and level of ATF4 expression are critical parameters determining cell fate (11). It was reported that even minor disturbances of ATF4 function contribute to serious pathologies (12). Furthermore, persistent overactivation of ATF4 is linked to many cancers because of the continuous expression of adaptive genes that sustain the stress response (13). Recently, Krausel et al. (14) indicated that the ATF4 mediates endoplasmic reticulum stress-related podocyte injury, suggesting that ATF4 plays a critical role in the kidney diseases. However, the potential role of ATF4 on tubulointerstitial fibrosis has not been established.

Besides endoplasmic reticulum stress, emerging evidence indicated that ATF4 plays an important role in regulating metabolic homeostasis (15). Accordingly, Liu et al. identified that ATF4 knockdown in macrophage impairs glycolysis and mediates immune tolerance (16). Moreover, using multi-omics analysis, ATF4 was found to reprogram cellular metabolism through activation of the integrated stress response (17). Thus, we speculated that ATF4 may be involved in the tubulointerstitial fibrosis by regulating metabolism.

Here, the potential role of ATF4 in tubulointerstitial fibrosis was explored from a metabolic perspective. Interestingly, we found that tubular ATF4 was significantly induced in mice with UUO. Further, we indicated that ATF4 played a crucial role in promoting renal tubulointerstitial fibrosis. Mechanistically, ATF4 functioned as a crucial glycolytic activator through transcriptional regulation of hexokinase II. Collectively, our findings not only represent novel insights into the pathogenesis of tubulointerstitial fibrosis but also provide a promising therapeutic targeting for the delaying the progression of CKD.

## 2 Materials and methods

### 2.1 Mice

All animal experiments were conducted using male C57BL/6J mice (6–8 weeks old; Vital River Laboratory Animal Technology Co., Ltd., Beijing, China). Mice were maintained in a controlled animal facility at 19-21 °C with 45%-65% relative humidity, under a 12-hour light/dark cycle. Animals were randomly allocated to experimental groups. Tubulointerstitial fibrosis was induced using a well-established unilateral ureteral obstruction (UUO) model, in which the right ureter was ligated just below the renal pelvis. Sham-operated mice underwent identical surgical procedures without ureteral ligation. On day 10 post-UUO, mice were euthanized under general anesthesia, and kidneys were collected for analysis. Kidney tissue sections were subjected to Periodic Acid-Schiff, Sirius Red staining and Masson’s trichrome staining. Software “ImageJ 1.53t” was used to randomly calculate the proportion of fibrotic area in the visual field under Masson staining across 5 fields for each mouse, followed by inter-group comparison.

Lentiviruses carrying ATF4 knockdown constructs or nonsense controls (NC) in the GVC396 vector were obtained from Genechem (Shanghai, China). Lentivirus-mediated gene delivery to the kidneys was achieved via tail vein injection (2 × 10^9^ TU per mouse). Three days after UUO surgery, lentiviral administration was performed, and animals were sacrificed on day 10 (n = 6 per group).

To further delineate the role of glycolysis in renal tubulointerstitial fibrosis, UUO mice were administered intraperitoneal injections of 2-deoxy-D-glucose (2-DG, 500 mg/kg; Sigma-Aldrich, USA) to inhibit glycolytic activity, and were sacrificed 10 days thereafter (n = 6 per group).

All animal procedures were approved by the Animal Ethics Committee of Southeast University.

### 2.2 Cell culture and treatment

The human tubular epithelial cell line HK-2 (Cat. CRL-2190) was obtained from the American Type Culture Collection. Cells with passage 7–20 were maintained in DMEM/Ham’s-F12 medium (HyClone, GE Healthcare Life Sciences, UT, USA) supplemented with 10% fetal bovine serum and 1% penicillin-streptomycin (Gibco, Grand Island, NY, USA) in a humidified incubator at 37 °C with 5% CO_2_ and 21% O_2_. HK-2 cells were seeded into six-well plates and cultured in complete medium until reaching approximately 80% confluence, after which the medium was replaced with serum-free medium for 12 hours. Cells were then exposed to transforming growth factor-β1 (TGF-β1, 10 ng/mL, Cat 100-21; PeproTech, United States) for 48 hours to induce fibrogenesis.

To assess the impact of ATF4 on fibrogenesis, HK-2 cells were transduced with ATF4 knockdown lentiviruses or ATF4 overexpression lentiviruses. In addition, to explore the role of hexokinase II in fibrogenesis, HK-2 cells were transduced with hexokinase II overexpression lentiviruses. Three days later, the cells were treated with TGF-β1 (10 ng/mL) for 48 hours, after which they were harvested for subsequent analyses.

### 2.3 Protein extraction and western blotting

Kidney cortex tissues and cultured cells were lysed in RIPA buffer supplemented with protease and phosphatase inhibitor cocktails (Cell Signaling Technology). Protein concentrations were determined using the Pierce BCA assay (Beyotime, Beijing, China). Equal amounts of protein (35-50 μg) were separated on 10% SDS-PAGE gels and transferred to polyvinylidene fluoride membranes (Millipore, MA, USA). After blocking with NcmBlot blocking buffer (NCM Biotech, Suzhou, China), membranes were incubated overnight at 4 °C with primary antibodies. Subsequently, membranes were incubated for 2 hours at room temperature with horseradish peroxidase conjugated anti-rabbit IgG (1:3000; 7076S, Cell Signaling Technology, USA), and immunoreactive bands were visualized using an enhanced chemiluminescence detection system (GE Healthcare, USA). The primary antibodies included rabbit anti-α-SMA (ab5694, Abcam, UK), rabbit anti-fibronectin (ab2413, Abcam, UK), rabbit anti-ATF4 (11815S, Cell Signaling Technology, USA), rabbit anti-hexokinase II (ab209847, Abcam, UK), and rabbit anti-β-actin (AY0573, Abways, China).

### 2.4 Lactate concentration

Lactate levels in lysed kidney cortex tissues and HK-2 cells were determined using a Lactate Assay Kit (Sigma-Aldrich) following the manufacturer’s instructions.

### 2.5 Oxygen consumption rate and extracellular acidification rate measurement

Seahorse Bioscience XFp Extracellular Flux Analyzer was utilized to quantify the dissolved oxygen and pH changes in the medium immediately surrounding the cell cultures. On the day of OCR assessment, the growth medium was replaced with assay medium composed of XF minimal DMEM supplemented with 10 mM D-glucose, 1 mM sodium pyruvate, and 2 mM L-glutamine, and incubated for 1 hour at 37°C in a CO_2_-free environment. OCR measurements were recorded at baseline (resting state) and following sequential injections of oligomycin (1 μM), carbonyl cyanide-p-trifluoromethoxyphenylhydrazone (FCCP; 1 μM), and a combination of rotenone and antimycin A (0.5 μM).

For the ECAR measurements, the growth medium was substituted with XF minimal DMEM containing 2 mM L-glutamine and incubated for 1 hour at 37°C in the absence of CO_2_. ECAR values were recorded at baseline and following sequential injections of D-glucose (10 mM), oligomycin (1 μM), and 2-deoxy-D-glucose (50 mM).

### 2.6 Total RNA isolation and quantitative real-time PCR

Total RNA was isolated from mouse renal cortex tissues and cultured cells using RNAiso Plus (Vazyme, Nanjing, China) following the manufacturer’s protocol. Complementary DNA was synthesized via reverse transcription using HiScript III RT SuperMix (Vazyme). Quantitative real-time PCR was performed on a 7300 Real-Time PCR System (Applied Biosystems, USA) employing 2× ChamQ SYBR qPCR Master Mix (Vazyme). Gene expression levels were normalized to β-actin, and all primer sequences used are detailed in **Table 1**.

**Table 1:** The primers.

Species	Gene	Forward primer (5S’-3’)	Reverse primer (5’-3’)
Mus	*Ccl2*	TAAAAACCTGGATCGGAACCAAA	GCATTAGCTTCAGATTTACGGGT
Mus	*Tnf-α*	CTGAACTTCGGGGTGATCGG	GGCTTGTCACTCGAATTTTGAGA
Mus	*Il-1β*	GAAATGCCACCTTTTGACAGTG	TGGATGCTCTCATCAGGACAG
Mus	*α-Sma*	CAGCAAACAGGAATACGACGAA	AACCACGAGTAACAAATCAAAGC
Mus	*Fn*	GCAAGAAGGACAACCGAGGAAA	GGACATCAGTGAAGGAGCCAGA
Mus	*Atf4*	CCACCAGACAATCTGCCTTC	CTAGCTCCTTACACTCGCCA
Mus	*Hk2*	TGATCGCCTGCTTATTCACGG	AACCGCCTAGAAATCTCCAGA
Mus	*β-actin*	GGCTGTATTCCCCTCCATCG	CCAGTTGGTAACAATGCCATGT
Homo	*Ccl2*	CAGCCAGATGCAATCAATGCC	TGGAATCCTGAACCCACTTCT
Homo	*Tnf-α*	GAGGCCAAGCCCTGGTATG	CGGGCCGATTGATCTCAGC
Homo	*Il-1β*	ATGATGGCTTATTACAGTGGCAA	GTCGGAGATTCGTAGCTGGA
Homo	*α-Sma*	ATGGTGATGACCTGCCCGTCT	ACTGAGCGTGGCTACTCCTTCG
Homo	*Fn*	GGAGCAAATGGCACCGAGATA	GAAGTGGGACCGTCAGGGAGA
Homo	*Atf4*	GGTTCTCCAGCGACA AGG	TCTCCAACATCCAATCTGTCC
Homo	*Hk2*	GAGCCACCACTCACCCTACT	CCAGGCATTCGGCAATGTG
Homo	*β-actin*	GGACCTGACCTGCCGTCTAG	GTAGCCCAGGATGCCCTTGA

Ccl2, C-C motif ligand 2; Tnf-α, tumor necrosis factor-α; Il-1β, interleukin-1β; α-Sma, α-smooth muscle actin; Fn, fibronectin; Atf4, activating transcription factor 4; Hk2, hexokinase II.

### 2.7 Luciferase reporter assay

The plasmids, including the pGL3-HRE-HK2-Luc luciferase reporter and ATF4 cloned into the pSD11 vector, were obtained from GenePharma (Shanghai, China). These plasmids were transfected into HK-2 cells using Lipofectamine 3000 (Invitrogen Corp., Carlsbad, CA, USA) according to the manufacturer’s protocol. The luciferase reporter assay was conducted following established procedures as previously described (18).

### 2.8 Immunohistochemical staining

Kidneys were fixed in 4% formaldehyde and embedded in paraffin. Paraffin-embedded sections were incubated overnight at 4 °C with primary antibodies against ATF4 (11815S, Cell Signaling Technology, USA) and hexokinase II (ab209847, Abcam, UK). Immunostaining was subsequently performed using a streptavidin-peroxidase detection system (Maixin Technology Co., Ltd., Fuzhou, China) following the manufacturer’s instructions. Software “ImageJ 1.53t” was used to randomly calculate the proportion of DAB staining areas in the visual field under across 5 fields for each mouse, followed by inter-group comparison.

### 2.9 Bioinformatics analysis

The promoter region of the hexokinase II gene was retrieved from the NCBI database (https://www.ncbi.nlm.nih.gov). Potential transcription factor binding sites within this region were predicted using JASPAR2024 (https://jaspar.elixir.no).

### 2.10 Chromatin immunoprecipitation assay

Chromatin immunoprecipitation (ChIP) assays were conducted using the Simple ChIP Plus Enzymatic Chromatin IP Kit with Magnetic Beads (#9003, Cell Signaling Technology, USA) following established protocols. Immunoprecipitation was carried out using an anti-ATF4 antibody (11815S, Abcam, UK) or normal IgG as a negative control. The precipitated DNA fragments were analyzed by PCR using primers specific to the hexokinase II promoter region.

### 2.11 Statistical analysis

Data are expressed as mean ± standard deviation (SD). For comparisons between two groups, either a two-tailed unpaired Student’s t-test or Mann-Whitney U test was utilized. When evaluating differences among multiple groups, one-way analysis of variance (ANOVA) followed by Dunnett’s or Bonferroni correction *post hoc* test was performed. Statistical significance was defined as p < 0.05. All analyses were conducted using GraphPad Prism 8.0 (GraphPad, MA, USA).

## 3 Results

### 3.1 Upregulated tubular ATF4 is associated with tubulointerstitial fibrosis

**Figure 1:**
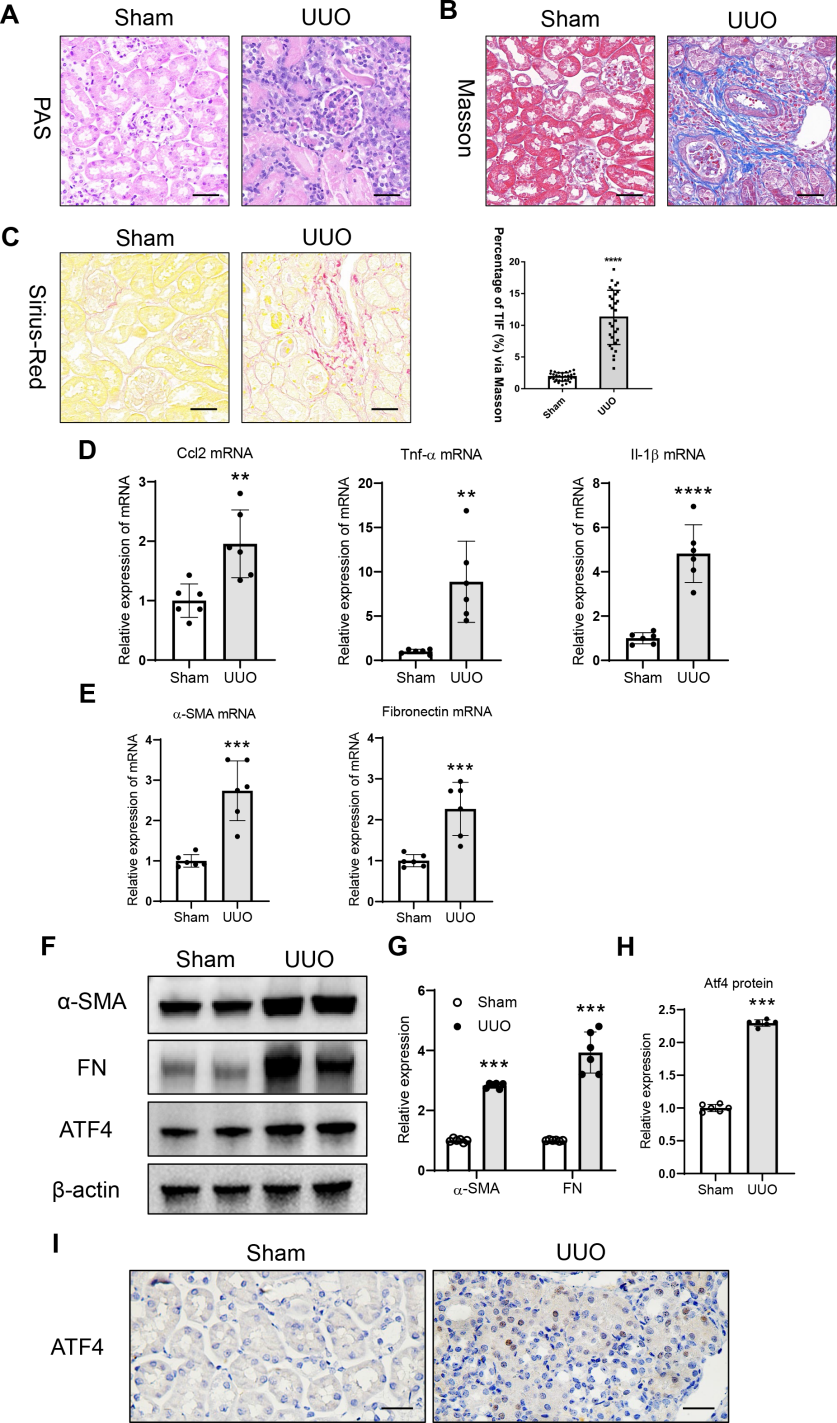
Tubular ATF4 expression are observed in the kidney with unilateral ureteral obstruction. **(A)**. Histological changes (periodic Acid-Schiff [PAS] staining). Bar = 50 μm. **(B)**. Histological changes of Sirius Red Staining. Bar = 50 μm. **(C)**. Histological changes of Masson’s trichrome staining. Bar = 50 μm. **(D)**. mRNA expression of inflammatory factors (C-C motif ligand 2 [CCL2], tumor necrosis factor-α [TNF-α], and interleukin-1β [IL-1β]) in the kidney with UUO assessed by quantitative real-time polymerase chain reaction (qRT-PCR). **(E)**. mRNA expression of fibrosis markers (alpha-smooth muscle actin [α-SMA] and fibronectin). **(F)**. Western blotting analysis of fibrosis markers and ATF4 expression in the kidney with UUO. **(G, H)**. Densitometric analysis of fibrosis markers and ATF4 expression in kidney. **(I)**. Representative images of ATF4 expression in the kidney from UUO mice assessed by immunohistochemistry. Bar = 50 μm. Data are mean ± SD for groups of 6 mice. **P < 0.01, ***P < 0.001, ****P < 0.0001 versus sham.

In the present study, unilateral ureteral obstruction-induced kidney injury was studied for renal tubulointerstitial fibrosis. Histologically, tubular epithelial cell injury and protein casts were found in the UUO-treated kidneys (**Figure 1A**). Tubulointerstitial fibrosis was observed in the UUO-treated kidneys, as evidenced by the Sirius Red staining and Masson’s trichrome staining (**Figures 1B, C**). Concomitantly, there were significant increases in mRNA expression of renal inflammatory cytokines (C-C motif ligand 2 [CCL2], tumor necrosis factor-α [TNF-α], and interleukin-1β [IL-1β]) and fibrosis markers (α-SMA and fibronectin) (**Figures 1D, E**). Meanwhile, protein expressions of fibrosis markers (α-SMA and fibronectin) were also detected by Western blotting (**Figures 1F, G**). Moreover, the increased in ATF4 mRNA and protein expression were found (**Figures 1F, H**; **Supplementary Figure 1**). Immunohistochemistry analysis further revealed that expression level of ATF4 was significantly increased, with this upregulation predominantly localized in the tubular epithelial cells of UUO-treated kidneys (**Figure 1I**). Thus, the induction of tubulointerstitial fibrosis was strongly associated with the expression of tubular ATF4, implying a potential link between tubular ATF4 expression and renal tubulointerstitial fibrosis responses in the UUO kidney.

### 3.2 ATF4 knockdown attenuates renal tubulointerstitial fibrosis

**Figure 2:**
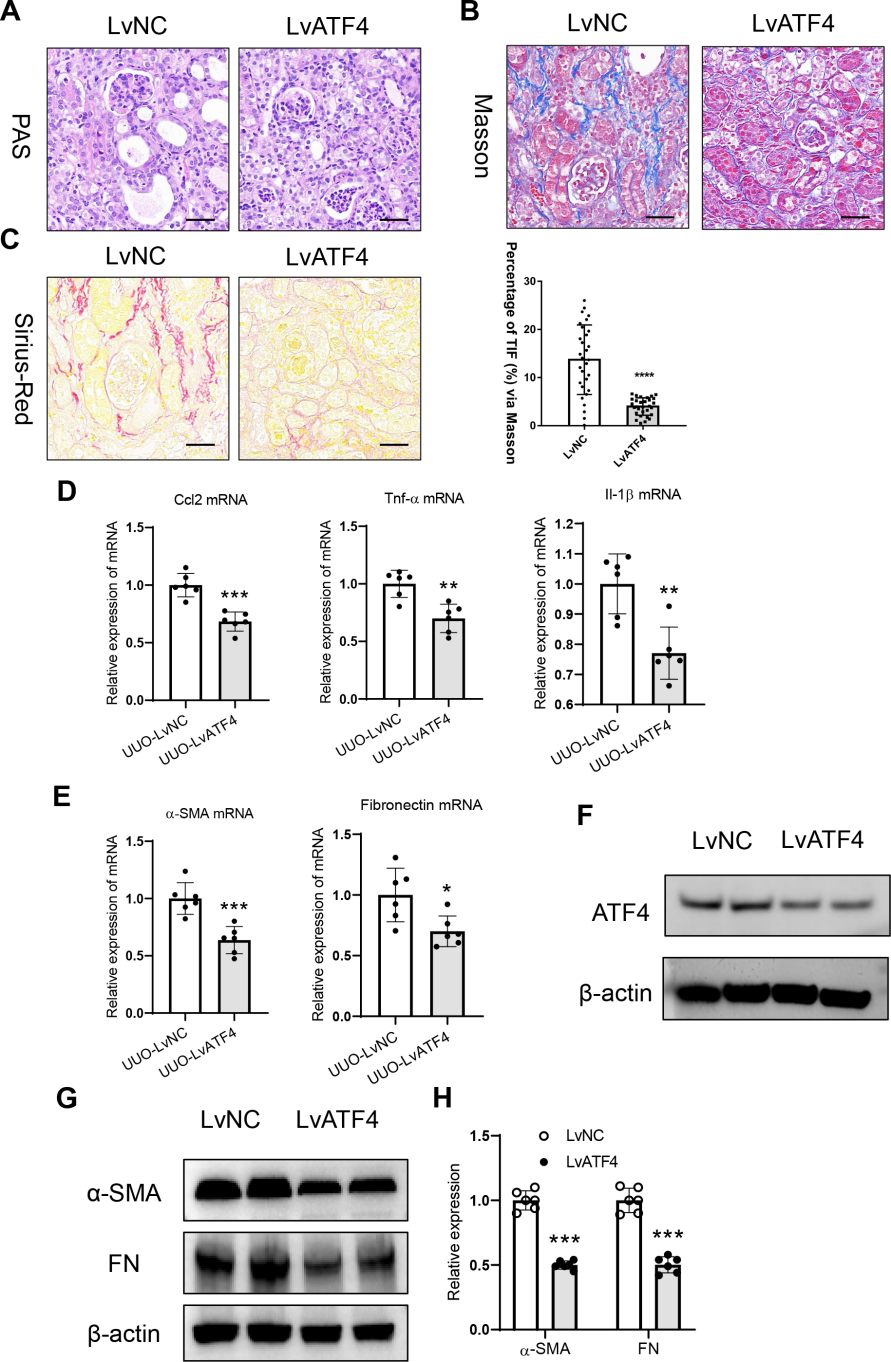
ATF4 knockdown represses renal tubulointerstitial fibrosis in unilateral ureteral obstruction-induced kidney injury. **(A)**. Histological changes in the UUO kidney from mice with ATF4 knockdown (PAS staining). Bar = 50 μm. **(B)**. Histological changes of Sirius Red Staining in the UUO kidney from mice with ATF4 knockdown. Bar = 50 μm. **(C)**. Histological changes of Masson’s trichrome staining in the UUO kidney from mice with ATF4 knockdown. Bar = 50 μm. **(D)**. mRNA expression of inflammatory factors in the UUO kidney with ATF4 knockdown. **(E)**. mRNA expression of fibrosis markers in the UUO kidney with ATF4 knockdown. **(F)**. ATF4-knockdown lentivirus successfully reduced ATF4 protein expression in UUO kidneys. **(G)**. Western blotting analysis of fibrosis markers in the UUO kidney with ATF4 knockdown. **(H)**. Densitometric analysis of fibrosis markers expression in the UUO kidney with ATF4 knockdown. Data are mean ± SD for groups of 6 mice. *P < 0.05, **P < 0.01, ***P < 0.001, ****P < 0.0001 versus LvNC.

To determine the role of ATF4 in tubulointerstitial fibrosis, ATF4-knockdown lentivirus was administered via the tail vein of mice with UUO. Interestingly, compared with scrambled negative control-injected mice, the ATF4 knockdown significantly ameliorated tubular epithelial cells injury and reduce protein casts (**Figure 2A**). Meanwhile, markedly alleviated tubulointerstitial fibrosis was found in the UUO-treated kidneys with ATF4-knockdown lentivirus administration (**Figures 2B, C**). Furthermore, the mRNA expression of inflammatory factors (MCP-1, TNF-α, and IL-1β) and fibrosis markers were similarly attenuated in this group (**Figures 2D, E**). Moreover, Western blotting was employed to detect the protein expression levels of ATF4 and fibrosis markers (**Figures 2F–H**). The results demonstrated that the ATF4-knockdown lentivirus not only successfully reduced ATF4 protein expression but also led to a significant decrease in the levels of fibrosis-related proteins. This finding further confirms the regulatory role of ATF4 in the progression of renal tubulointerstitial fibrosis, as the suppression of ATF4 directly correlates with the downregulation of key fibrotic proteins.

### 3.3 Knockdown of ATF4 ameliorates fibrogenesis of tubule epithelial cell

**Figure 3:**
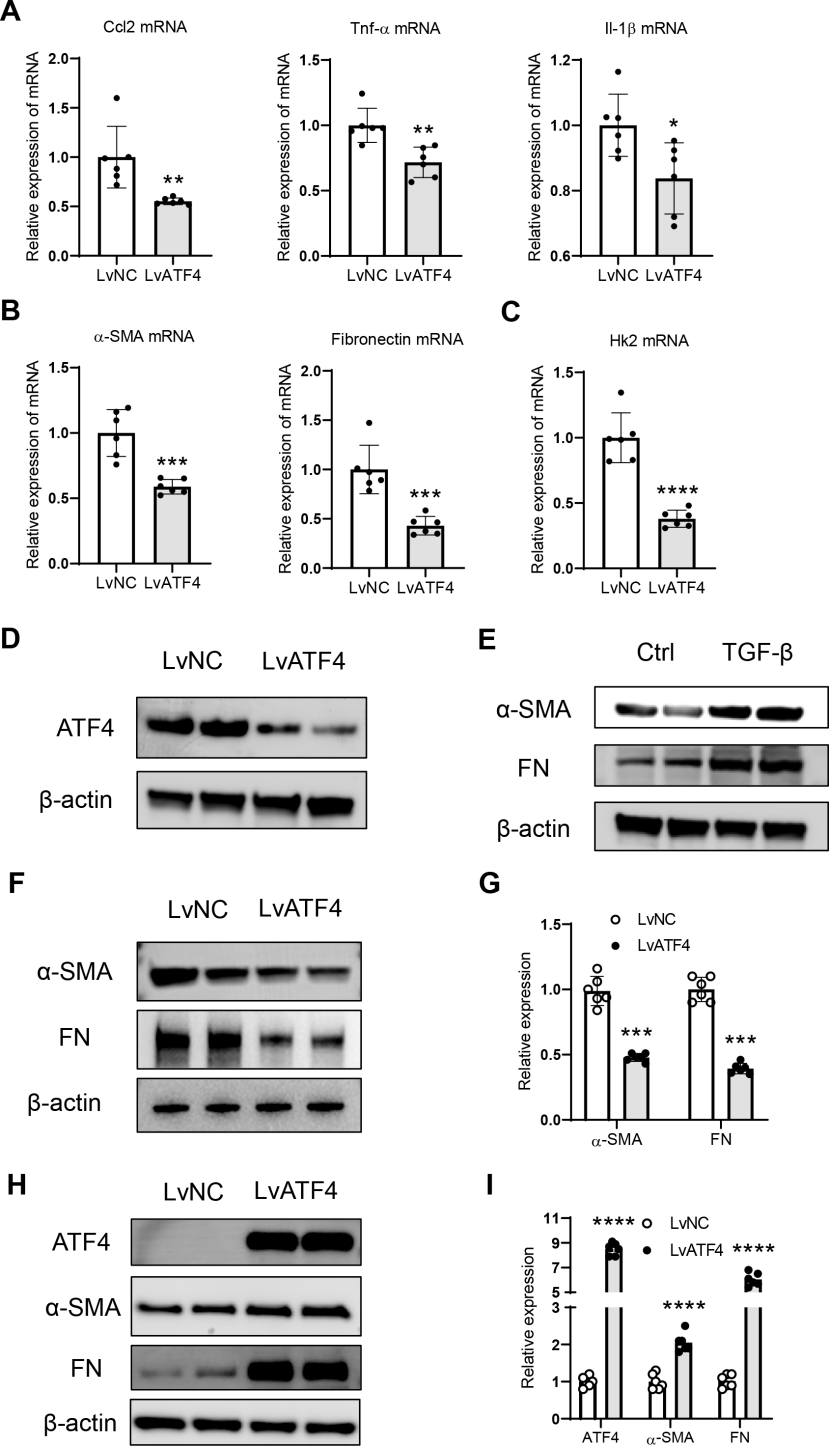
Knockdown of ATF4 inhibits fibrogenesis of tubule epithelial cell. **(A)**. mRNA expression of inflammatory factors in the HK-2 cells with ATF4 knockdown. **(B)**. mRNA expression of fibrosis markers in the HK-2 cells with ATF4 knockdown. **(C)**. mRNA expression of hexokinase II in the HK-2 cells with ATF4 knockdown. **(D)**. ATF4-knockdown lentivirus successfully reduced ATF4 protein expression in HK-2 cells. **(E)**. TGF-β could induce HK-2 cells to produce increased amounts of fibrotic proteins. **(F)** Western blotting analysis of fibrosis markers in the HK-2 cells with ATF4 knockdown. **(G)**. Densitometric analysis of fibrosis markers expression in the HK-2 cells with ATF4 knockdown. **(H)** Western blotting analysis of ATF4 and fibrosis markers expression in the HK-2 cells with ATF4 overexpression. **(I)**. Densitometric analysis of ATF4 and fibrosis markers expression in the HK-2 cells with ATF4 overexpression. Data are mean ± SD (n=6). *P < 0.05, **P < 0.01, ***P < 0.001, ****P < 0.0001 versus LvNC.

To directly investigate the functional effects of ATF4 on fibrogenesis in tubule epithelial cells, we knocked down ATF4 expression by subjecting tubular epithelial cells to ATF4-knockdown lentivirus *in vitro*. As expected, the mRNA expression of inflammatory factors (MCP-1, TNF-α, and IL-1β) and fibrosis markers were significantly attenuated in the tubule epithelial cell with ATF4 knockdown (**Figures 3A, B**). Furthermore, we also indicated that the expression of hexokinase II mRNA was significantly decreased in this group (**Figure 3C**), implying that the glycolysis was inhibited. Using the Western blotting method, we found that the ATF4-knockdown lentivirus could downregulate the expression level of ATF4 protein in tubule epithelial cells (**Figure 3D**). In addition, stimulation with TGFβ1 was shown to increase the expression of fibrotic proteins (**Figure 3E**). Furthermore, we observed that the ATF4-knockdown lentivirus could reduce the expression of fibrotic proteins (**Figures 3F, G**), while the ATF4-overexpression could promote the expression of fibrotic proteins (**Figures 3H, I**). Collectively, these findings indicate that knockdown of ATF4 ameliorates fibrogenesis of tubule epithelial cell directly.

### 3.4 Glycolysis is involved in the ATF4-mediated tubulointerstitial fibrosis

**Figure 4:**
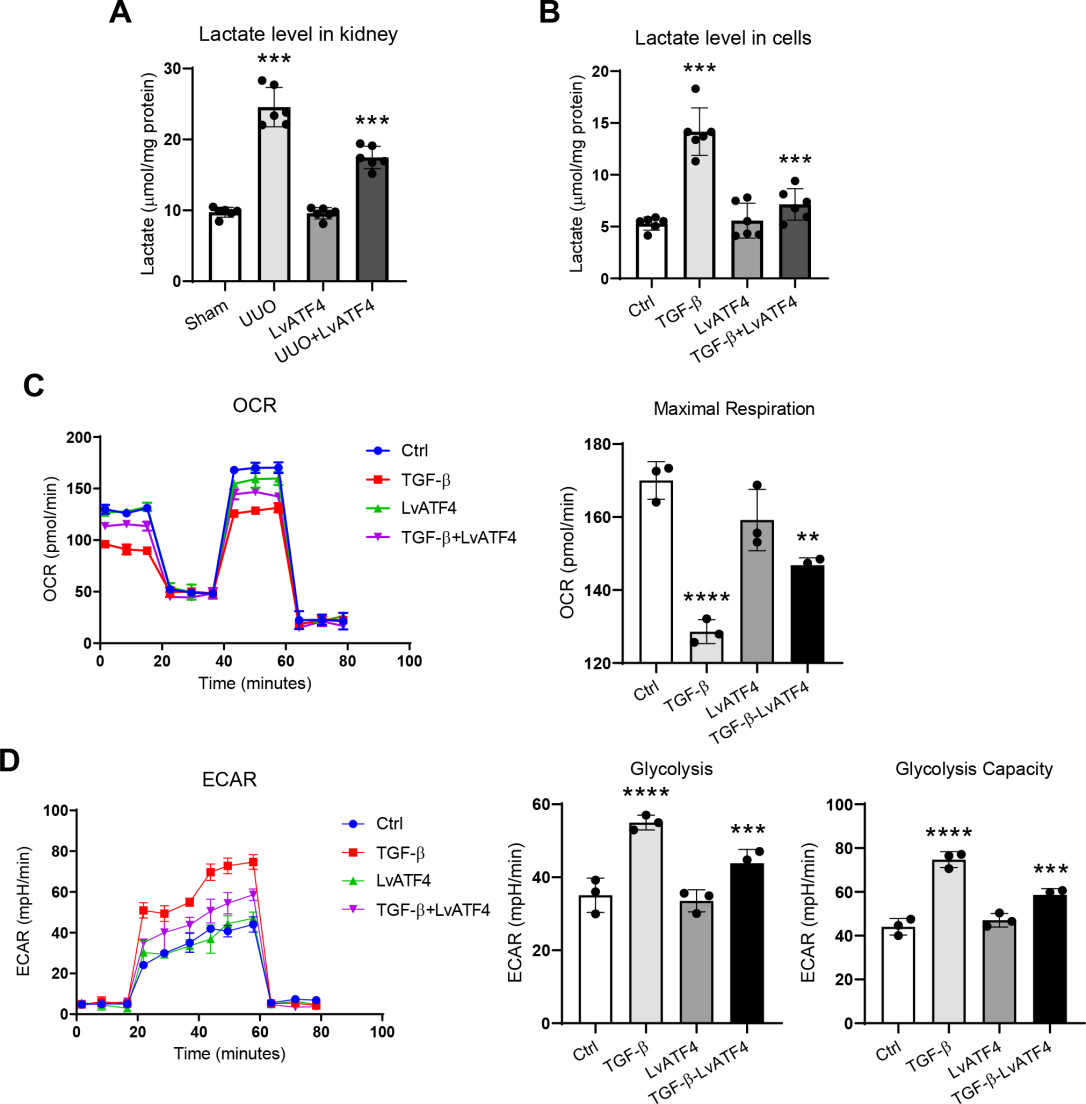
Glycolysis is associated with tubular ATF4 activation in the kidney with unilateral ureteral obstruction. **(A)**. Lactate levels in the kidney cortex; **(B)**. Lactate levels in the HK-2 cells; **(C)**. Mitochondrial OCR measured in ATF4-knowdown HK-2 cells treated with TGF-β1 for 48 hours. **(D)**. Mitochondrial ERAC measured in ATF4-knowdown HK-2 cells treated with TGF-β1 for 48 hours **P < 0.01, ***P < 0.001, ****P < 0.0001.

Then, the concentration of lactate, an end product of glycolysis, was assessed. Interestingly, we found that the concentration of lactate was markedly increased in the kidney with UUO. While the elevated lactate levels were significantly reversed in the renal tissues of ATF4-knockdown mice (**Figure 4A**). Simultaneously, the consistent pattern was observed in renal tubular cells *in vitro* (**Figure 4B**). Then, to measure the metabolism pathways in HK-2 cells, we quantified mitochondrial OCR and ECAR. As expect, we found that knockdown of ATF4 increased mitochondrial OCR (**Figure 4C**) in HK-2 cells with TGF-β1 treatment. Additionally, a similar pattern on ECAR was observed (**Figure 4D**). These data demonstrated that ATF4 contributes to the regulation of glycolysis.

### 3.5 Glycolysis inhibition ameliorates renal tubulointerstitial fibrosis

**Figure 5:**
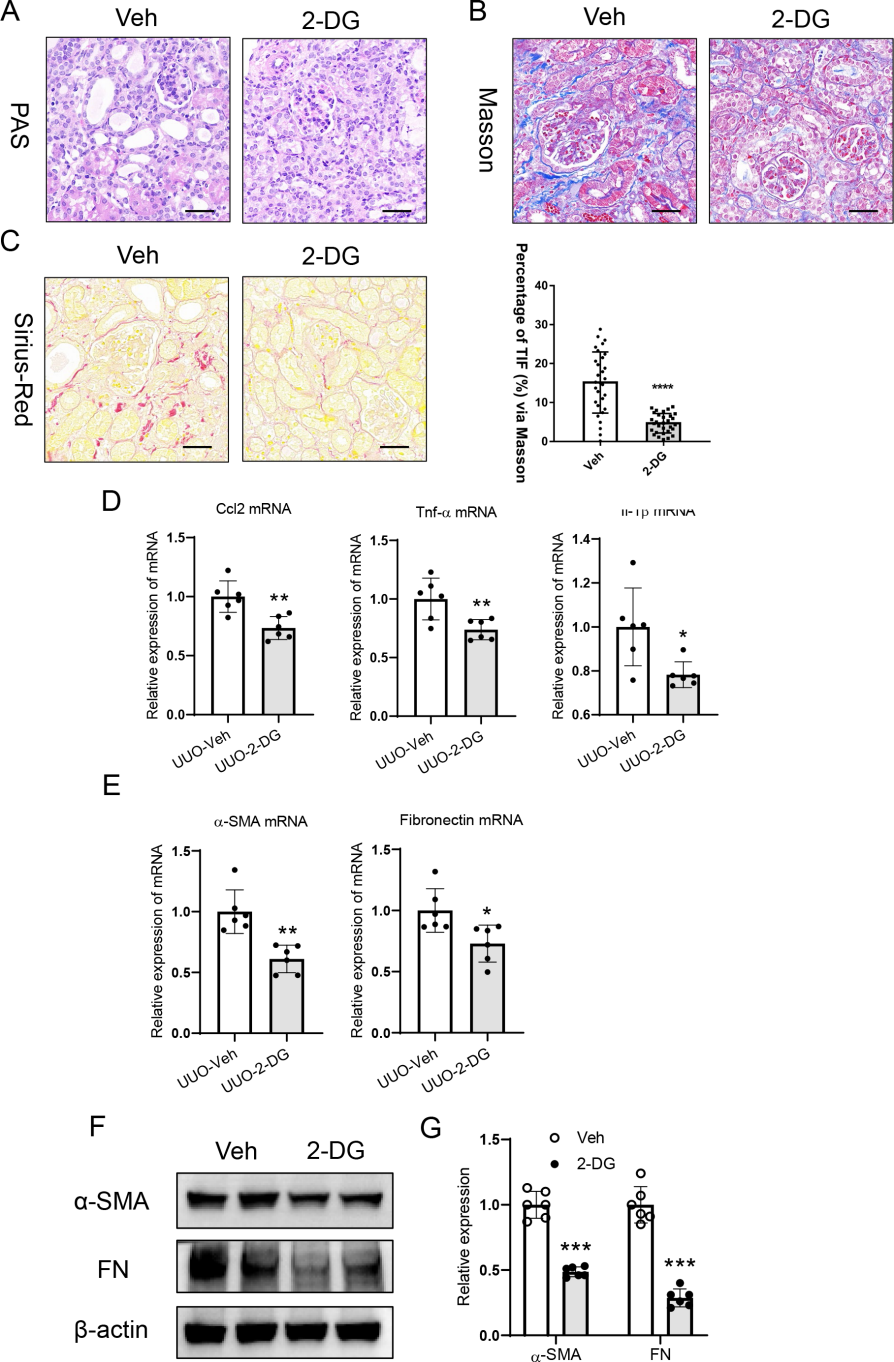
Glycolysis inhibition ameliorates renal tubulointerstitial fibrosis. **(A)**. Histological changes in the UUO kidney with 2-DG treatment (PAS staining). Bar = 50 μm. **(B)**. Histological changes of Sirius Red Staining in the UUO kidney from mice with 2-DG treatment. Bar = 50 μm. **(C)**. Histological changes of Masson’s trichrome staining in the UUO kidney from mice with 2-DG treatment. Bar = 50 μm. **(D)**. mRNA expression of inflammatory factors in the UUO kidney with 2-DG treatment. **(E)**. mRNA expression of fibrosis markers in the UUO kidney with 2-DG treatment. **(F)**. Western blotting analysis of fibrosis markers in the UUO kidney with 2-DG treatment. **(G)**. Densitometric analysis of fibrosis markers expression in the UUO kidney with 2-DG treatment. Data are mean ± SD for groups of 6 mice. *P < 0.05, **P < 0.01, ***P < 0.001, ****P < 0.0001 versus Vehicle.

To investigate the role of glycolysis in tubulointerstitial fibrosis, the UUO mice were administered intraperitoneally with glycolysis specific inhibitor 2-DG. Interestingly, compared with vehicle control-injected mice, glycolysis inhibition significantly ameliorated tubular epithelial cells injury and reduce protein casts (**Figure 5A**). Meanwhile, markedly alleviated tubulointerstitial fibrosis was observed in the UUO-treated kidneys with 2-DG administration (**Figures 5B, C**). Furthermore, the mRNA expression of inflammatory factors (MCP-1, TNF-α, and IL-1β) and fibrosis markers were similarly attenuated in this group (**Figures 5D, E**). Moreover, westering blotting analysis revealed that glycolysis inhibitor 2-DG treatment significantly reduced fibrosis markers expression (**Figures 5F, G**). Thus, these data indicate that glycolysis is involved in the tubulointerstitial fibrosis.

### 3.6 ATF4 promotes renal tubulointerstitial fibrosis by transcriptionally regulating hexokinase II

**Figure 6:**
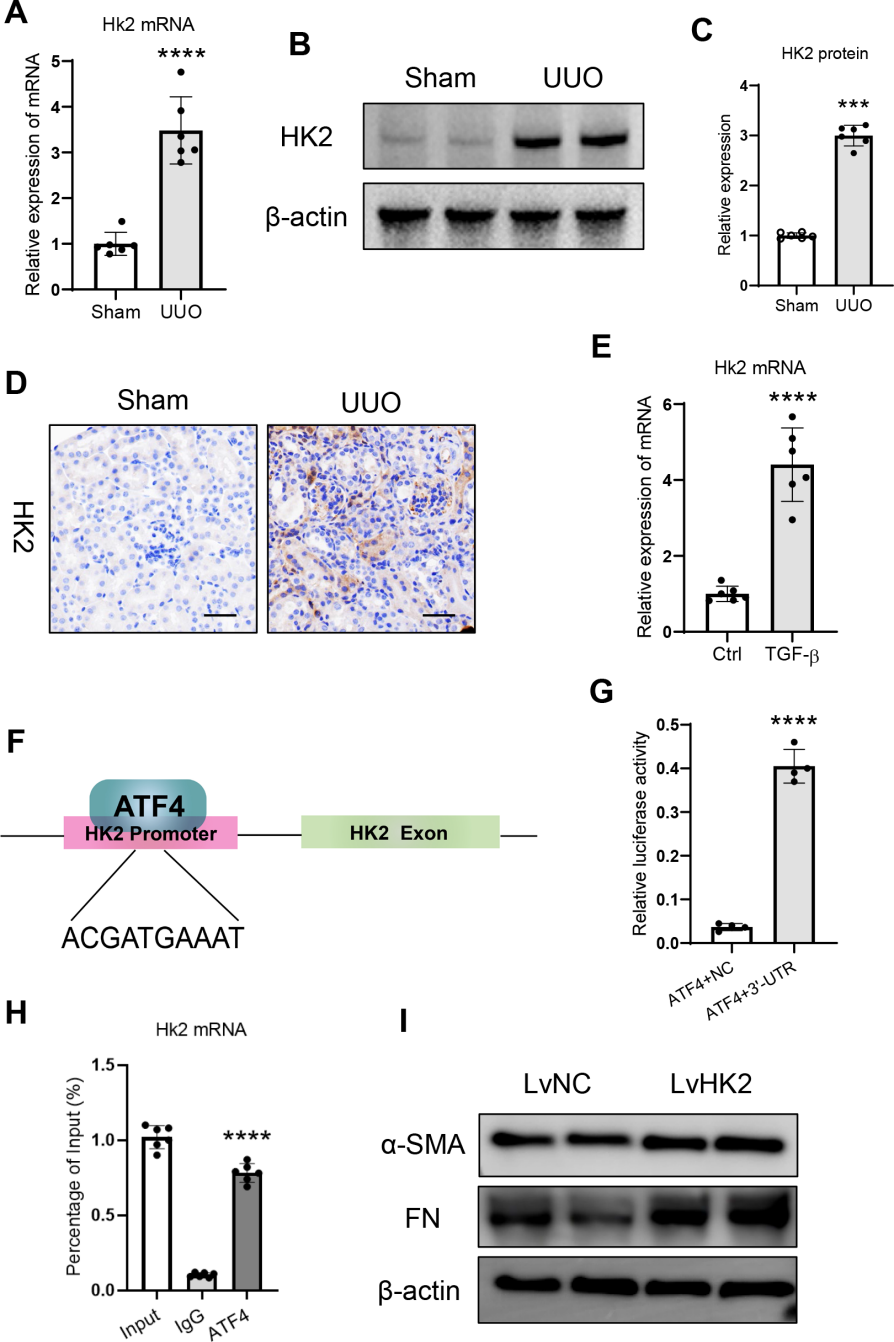
ATF4 transcriptionally regulates hexokinase II in renal tubulointerstitial fibrosis. **(A)**. mRNA expression of hexokinase II in the UUO kidney. **(B)**. Western blotting analysis of hexokinase II in the UUO kidney. **(C)**. Densitometric analysis of hexokinase II expression in the UUO kidney. **(D)**. Representative images of hexokinase II expression in the kidney from UUO mice assessed by immunohistochemistry. Bar = 50 μm. **(E)**. mRNA expression of ATF4 in the HK-2 cells with TGF-β1 administration. **(F)**. The potential target of ATF4 in the promoter of hexokinase II gene. **(G)**. Luciferase reporter assay was performed using constructs with hexokinase II-3’-UTR. **(H)**. Chromatin immunoprecipitation assay shows a binding of ATF4 to the hexokinase II promoter. **(I)**. Western blotting analysis of fibrosis markers in the ATF-knockdown cells with hexokinase II overexpression lentivirus treatment. Data are mean ± SD (n=6). ***P < 0.001, ****P < 0.0001 versus Sham or Control.

**Figure 7:**
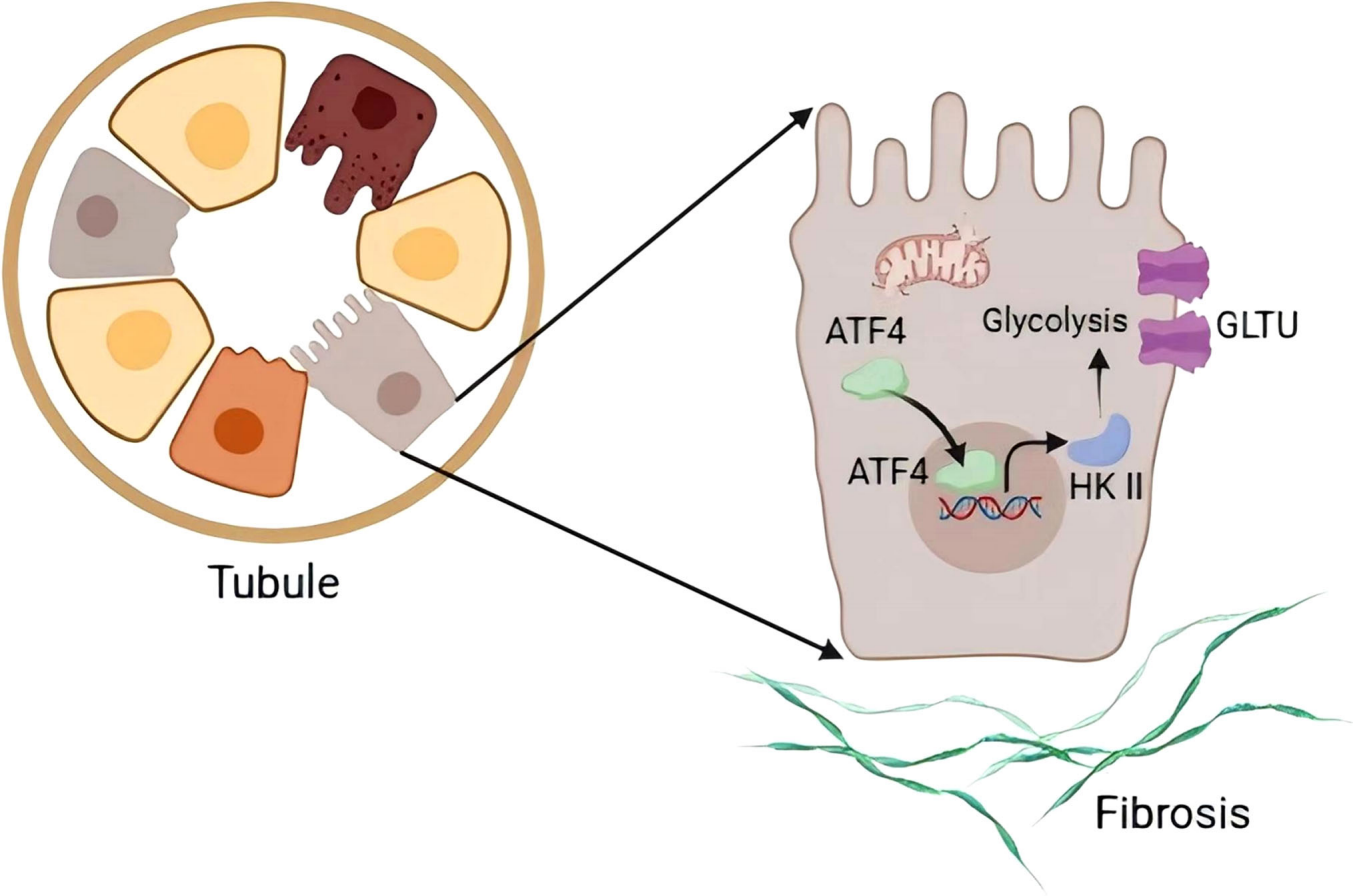
Schematic diagram showing the proposed molecular responses of activating transcription factor 4 signaling in renal tubulointerstitial fibrosis. During the progression of chronic kidney disease, tubular activating transcription factor 4 promotes tubulointerstitial fibrosis by activating hexokinase II-mediated glycolysis.

Then, the potential mechanism of ATF4 on glycolysis regulation was explored. It is well-known that hexokinase II is one of the most critical rate-limiting enzymes governing glycolysis. Therefore, we speculate that ATF4 may be involved in glycolysis by regulating hexokinase II. Interestingly, we found that the hexokinase II mRNA expression was markedly increased in the UUO-treated kidney (**Figure 6A**). Meanwhile, the protein expression of hexokinase II in the kidney followed a similar pattern (**Figures 6B, C**). Immunohistochemical staining revealed that hexokinase II was highly expressed in the cytoplasm of proximal tubular epithelial cells in UUO (**Figure 6D**). Further, to directly investigate the potential effects of ATF4 on hexokinase II, we subjected tubular epithelial cells to TGF-β1 *in vitro*. As shown in **Figure 6E**, the expression of hexokinase II mRNA was significantly increased in the HK-2 cells with TGF-β1 treatment. Using in silico analysis, we found the presence of a putative ATF4 binding motif located within the upstream of transcriptional starting sites of the hexokinase II locus (**Figure 6F**). Luciferase reporter assay showed that the activity of luciferase reporters was markedly increased in the group with ATF4 overexpression and hexokinase II-3’-UTR administration (**Figure 6G**), demonstrating that ATF4 could directly regulate hexokinase II expression. Additionally, chromatin immunoprecipitation assay showed that the binding of ATF4 was enriched in the hexokinase II promoter in tubular epithelial cells by TGF-β1 stimulation (**Figure 6H**), demonstrating the direct interaction between ATF4 and hexokinase II. Notably, overexpression of hexokinase-II could reverse the phenotype of HK-2 cells with ATF4-knockdown (**Figure 6I**). Thus, ATF4 transcriptionally regulates hexokinase II during the renal tubulointerstitial fibrosis (**Figure 7**).

## 4 Discussion

ATF4, a stress-induced transcription factor, plays a crucial role in the cellular response to multiple stresses. However, the potential role of ATF4 in tubulointerstitial fibrosis remains poorly understood. In the present study, we found that ATF4 was markedly increased in the UUO kidney. Further, we found that ATF4 played a crucial role in promoting renal tubulointerstitial fibrosis. Mechanistically, our data demonstrated that ATF4 functioned as a crucial glycolytic activator through transcriptional regulation of hexokinase II. Blockage of ATF4-glycolysis axis significantly ameliorated renal tubulointerstitial fibrosis. Therefore, our findings not only represent novel insights into the pathogenesis of tubulointerstitial fibrosis but also provide a promising therapeutic strategy for delaying the progression of CKD.

Due to their inherent characteristics, renal tubules are vulnerable to a variety of injuries. Notably, tubules are not only the victim of injury, but more importantly an instigator of kidney diseases (19). Accordingly, in response to injury, tubular epithelial cells undergo changes and function as inflammatory and fibrogenic cells, with the consequent production of various bioactive molecules that drive interstitial inflammation and fibrosis (20, 21). Especially, tubular epithelial cells play crucial roles in the progression of fibrosis by directly secreting various inflammatory factors and extracellular vesicle (22, 23). However, in the progression of renal fibrosis, the specific mechanism of orchestrating the fate of tubular cells remains poorly understood. Elucidating the exact molecular mechanism will help us gain novel insights into the pathophysiology of renal fibrosis and provide new therapeutic strategies to delay the progression of CKD.

ATF4, a master regulator of transcription of genes essential for stress, governs multiple signaling pathways, including autophagy, oxidative stress, inflammation, and translation, suggesting a multifaceted role of ATF4 in the progression of various pathologies (24, 25). However, the functional effects of ATF4 on kidney remains obscure. On one hand, Krausel et al. (14) indicated that the transcription factor ATF4 mediates endoplasmic reticulum stress-related slit diaphragm defects in podocyte diseases. On the other hand, it was found that ATF4 activation attenuates diabetic nephropathy by inducing autophagy and inhibiting apoptosis in podocyte through enhancing the expression of heme-oxygenase-1 (26). Thus, the functions of ATF4 appear to be complex under the condition of kidney diseases. In the present study, we for the first found that activation of tubular ATF4 promotes renal tubulointerstitial fibrosis in a UUO model. Given its physiological function, it is logical that tubular ATF4 activation promotes renal interstitial fibrosis.

Emerging evidence suggested that ATF4 orchestrates a response program associated with metabolic homeostasis. For instance, in a pressure overload-induced heart failure mice model, joint pathway analysis of transcriptomic and metabolomic data revealed that ATF4 preferably controlled oxidative stress and redox-related pathways (27). Therefore, we speculate that ATF4 may be involved in tubulointerstitial fibrosis through regulating metabolic pathways. Interestingly, we found that in the present study, tubular ATF4 was an important effector of glycolysis reprograming. Furthermore, our data demonstrated that ATF4 controlled glycolysis through regulation of hexokinase II. Mechanically, ATF4 bound to the promoter region of hexokinase II, which was consistent with previous results found by Liu et al. (16). Similarly, Zhou et al. recently reported that ATF4 promotes glycolysis under the condition of colorectal cancer (28). It should be note that ATF4 also played a crucial role in other metabolic pathways, such as lipid metabolism and amino acid metabolism (29, 30). However, whether ATF4-mediated other metabolic pathways were involved in tubulointerstitial fibrosis needs to be further explored.

In the present study, we also confirmed that blockade of ATF4-hexokinase 2-glycolysis pathway may serve as a novel therapeutic approach to ameliorate tubulointerstitial fibrosis. Of note, despite being in the set of kidney disease, we indicated a novel ATF4 regulatory mechanism, which is independent of the canonical signaling from either the unfolded protein response or the integrated stress response. In addition, as a key transcription factor, ATF4 expression can be regulated at transcriptional, translational, and post-translational levels (31–33). Thus, the regulatory dynamics of ATF4 under pathophysiological conditions are likely to be profoundly intricate. Previous researches have indicated that females may exhibit inherently higher baseline levels and biological activity of ATF4 compared with males (34, 35). The present study employed male mice as the experimental model, and thus the influence of ATF4 on glycolytic activity and renal fibrosis may differ across sexes. Additional limitations should also be acknowledged. In particular, evidence concerning ATF4 expression in patients with CKD remains unavailable. Moreover, the mechanistic basis is not fully established, as it has yet to be determined whether hexokinase II overexpression can counteract renal interstitial fibrosis induced by ATF4 silencing. These unresolved questions warrant rigorous investigation in future studies.

## 5 Conclusion

In summary, despite considerable interest and significant clinical implications, the mechanisms driving renal fibrosis remain incompletely understood. Here, we reveal that tubulointerstitial fibrosis correlates with elevated ATF4 expression in tubular epithelial cells. Moreover, our data indicated that ATF4 functions as a transcriptional regulator, directly modulating the expression of glycolytic genes. This uncovers a novel regulatory axis in the pathogenesis of renal fibrosis. Collectively, our findings offer fresh insights into tubulointerstitial fibrosis and highlight a potential therapeutic avenue for mitigating the progression of chronic kidney disease.

## Data Availability

The original contributions presented in the study are included in the article/**Supplementary Material**. Further inquiries can be directed to the corresponding authors.
