# Supplementary material for: ATF4 promotes renal tubulointerstitial fibrosis through hexokinase II-mediated glycolysis
Source: Front Immunol. 2025 Dec 17;16:1683249. doi: 10.3389/fimmu.2025.1683249 (PMC12753322; doi:10.3389/fimmu.2025.1683249)
Supplement: Supplementary file 1 [file DataSheet1.pdf]

Figure 1

Figure 1-4

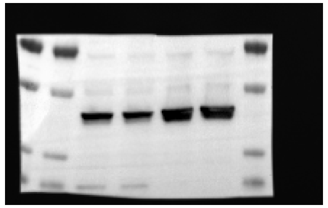

ctrl ctrl UUO UUO

aSMA

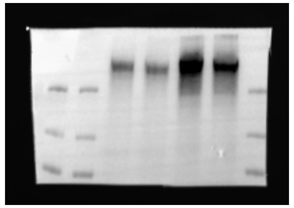

ctrl ctrl UUO UUO

FN

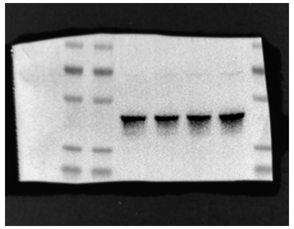

ctrl ctrl UUO UUO

Beta actin

“2025 01 22”

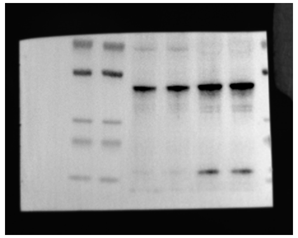

ctrl ctrl UUO UUO

ATF4

“2025 01 24 nwj”

Figure 2

Figure 2-4

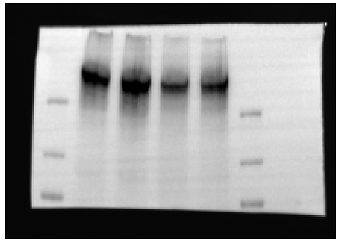

UUO; UUO; UUO+shATF4; UUO+shATF4

FN

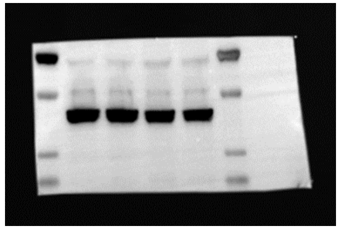

UUO; UUO; UUO+shATF4; UUO+shATF4

Beta actin

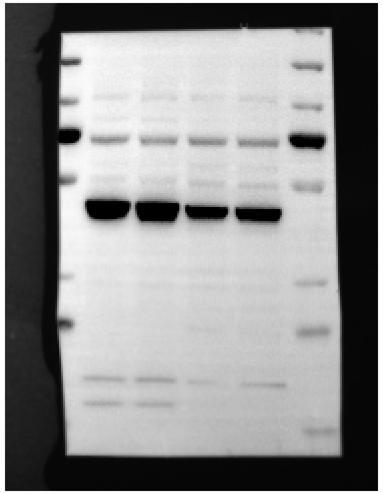

UUO; UUO; UUO+shATF4; UUO+shATF4

aSMA

Figure 3

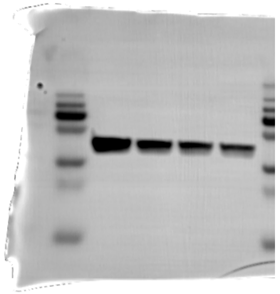

HK2 TGFb， TGFb， TGFb+shATF4， TGFb+shATF4

aSMA

“20250315”

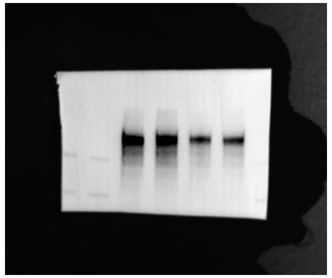

HK2 TGFb， TGFb， TGFb+shATF4， TGFb+shATF4

FN

“2025 01 22”

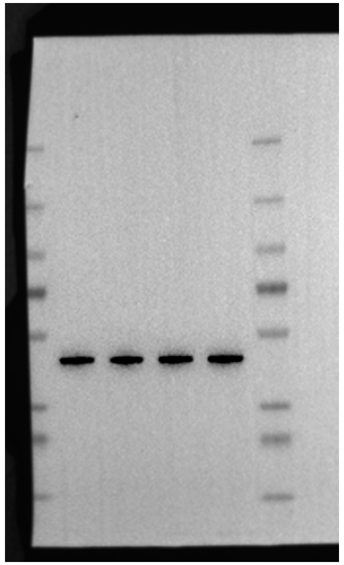

HK2 TGFb， TGFb， TGFb+shATF4， TGFb+shATF4

beta actin

“2025 01 24 nwj“”

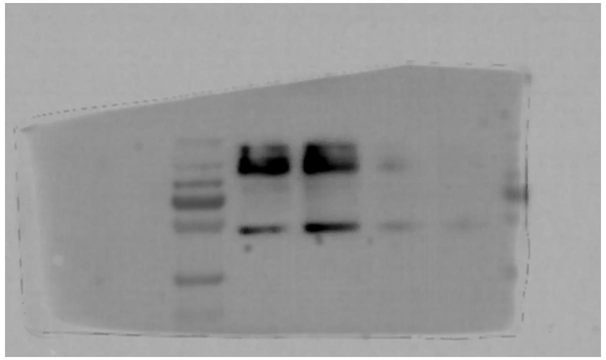

HK2 TGFb， TGFb， TGFb+shATF4， TGFb+shATF4

ATF4

“20250315“”

Figure 4

Figure 4-4

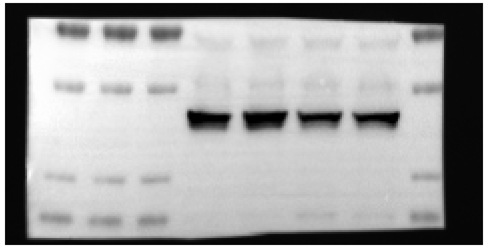

UUO; UUO; UUO+2DG; UUO+ 2DG

aSMA

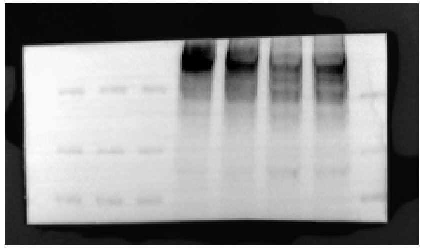

UUO; UUO; UUO+2DG; UUO+ 2DG

FN

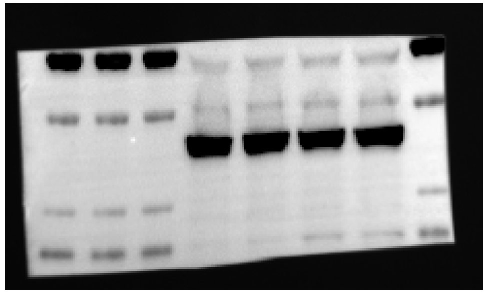

UUO; UUO; UUO+2DG; UUO+ 2DG

Beta actin

Figure 5

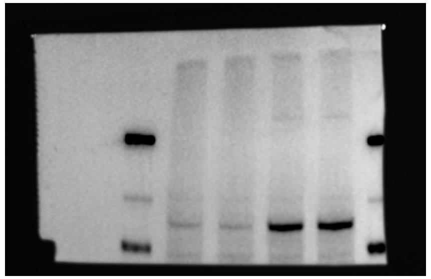

ctrl ctrl UUO UUO

HK2

“2025 01 24 nwj”

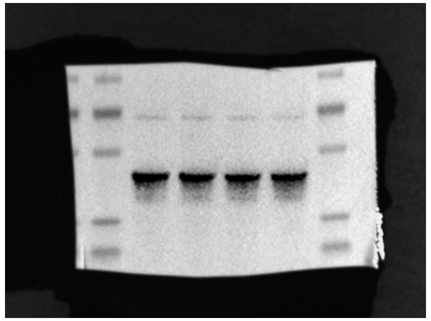

ctrl ctrl UUO UUO

Beta actin

“2025 01 22”
