# Supplementary material for: ATF4 promotes renal tubulointerstitial fibrosis through hexokinase II-mediated glycolysis
Source: Front Immunol. 2025 Dec 17;16:1683249. doi: 10.3389/fimmu.2025.1683249 (PMC12753322; doi:10.3389/fimmu.2025.1683249)
Supplement: Supplementary file 2 [file Table1.docx]

**Supplementary figure 1.** The expression of ATF4 mRNA was signiﬁcantly increased in the UUO-treated kidneys. a) The Atf4 mRNA expression in the UUO kidney was detected by RT-PCR. ***P*<0.01.
